# Dementia: Sustaining Self in the Face of Cognitive Decline

**DOI:** 10.3390/geriatrics1040025

**Published:** 2016-10-21

**Authors:** Caroline Hampson, Karen Morris

**Affiliations:** 1 Graduate School, University of Cumbria, Room 9, Gressingham Building, Bowerham Road, Lancaster LA1 3JD, UK; 2 Department of Health, Psychology and Social Studies, University of Cumbria, Fusehill Street, Carlisle CA1 2HH, UK; karen.morris@cumbria.ac.uk

**Keywords:** dementia, self, personhood, phenomenology

## Abstract

It is argued that the way in which we view a person with dementia can have a significant effect on the level of disability and wellbeing of the person. There is a divergence between a belief that the self disintegrates, leaving a non-person, and a belief that the self remains but is misplaced and can be maintained with the appropriate approach from others. This article seeks to examine the differing approaches to self and personhood in dementia care, and establish ways of approaching care for the person with dementia which may limit the extent of disability in the face of cognitive decline.

## 1. Introduction

Over the last 20 years, social psychology has become very prominent in the area of dementia care; proposing a notion of personhood which places the person with dementia at the centre of care, challenging and advancing alternatives to the understandings of dementia that were focussed on decay, decline and deficiency [1,2,3]. This alternative viewpoint on dementia is not a new one however, and has its background in phenomenology and the idea of ‘self’. This philosophical approach seeks to identify the “subtle and profound ways person–body–others–world are intimately *intertwined”* [4].

However, within the notion of ‘personhood’ there are two distinct opinions of the self and dementia that have arisen within the field of phenomenology. This article seeks to explore these opposing viewpoints and establish the most relevant position for healthcare professionals working in the field of dementia care.

## 2. Missing Person: Have You Seen Her?

“Her body might be with us, but her soul has left the room”.

[5]

The above quote is from a song by Rick Guard (released in 2012 to raise funds for the Alzheimer’s Society). The song is written about his mother, in which he describes her as a “missing person”. The song is a very moving and personal account of his experience with dementia, and interestingly describes a particular view of personhood held by Heidegger (1889–1976). This viewpoint is one which considers the human existence to be self-interpreting, with a temporal understanding of being and a dynamic engagement with the world through our bodies [6]. When a person has dementia, the cognitive dysfunction erodes our “being-in-the world” [6], which splinters the sense of being and therefore the sense of self. It is argued that the process of being in the world is disrupted, new experiences cannot be retained, and eventually the physical aspect of experiencing the world is impaired as cognition declines [6]. This approach to dementia considers that the person is undergoing a loss of self, which results in a non-person [7]. Indeed, Davis [6] states that dementia is:

“....a fraying of the self. Dementia effects the dismantling of the self until there is nothing left.”

[6]

This position assumes that the decline and loss of cognitive functions (e.g. consciousness, rationality, intentionality, memory, reciprocity, communication), causes a disintegration of personhood and an unbecoming of self [8,9,10,11,12]. If we adopt this standpoint and view people with dementia as a non-person, the ethical requirement to provide care and support is diminished [13]. This reduces care input so that only the physical needs of the person are met, and also risks that the life of the person is seen as meaningless [13,14]. Indeed, Singer [15,16] argues that a person with dementia gradually becomes and “empty husk” and that beyond a certain stage, the person is gone. He goes on to argue that a person with dementia should be euthanised in order to reduce the economic burden and the burden on caregivers [15,16]. However Singer’s utilitarian perspective has been heavily criticised, with some saying that Singer is ‘dangerous’, an intellectual psychopath, a ‘Nazi’ and morally wrong [17,18,19].

Herskovits [11], argued that this approach to the self in dementia stemmed from the medical model approach to care, and was nurtured to meet the needs of researchers, research institutions, and to solve clinical, practical and psychological issues. She argues that the discourse around the loss of self in dementia has created a vision of the person with dementia as a “dehumanised monster”, which generates a fear of growing older as people try to face their own “future potential monstrousness” should they develop dementia (p. 160, [11]).

From a practitioner perspective the perception that the self is eroded to nothing as dementia progresses is very difficult to accept, not least because people in the later stages of the illness are often still able to interact on a basic level with their environment, and are therefore demonstrating an ability to be in the world. Millett [7] argues that people with dementia continue to exist in the world of others (as parents, siblings, spouses, etc.), and therefore continue to have a value as a being. The cognitive losses do not make them of any less importance, and therefore an alternative view of the self in dementia should be sought. Interestingly, Herskovits [11] pointed out that this approach to the self in dementia is cognitively focussed, i.e. the self is inextricably linked to cognition, therefore any deterioration in cognitive ability leads to a diminishing of the self. This is different to other models of self in dementia care which were proposed towards the end of the nineteenth century, such as those proposed by Kitwood [20], Sabat and Harré [21] and Robertson [22] which place the focus on other factors for influencing the self and personhood on the person with dementia.

## 3. A Misplaced Being

“I am not at peace......I am in pieces”.

[23]

The above statement is from a lady with dementia. It is her response to being told to be at peace, and describes how a person with dementia experiences a fragmentation of self, rather than a loss of self [23]. While a very poignant statement, it also serves to demonstrate an alternative view of the self in dementia through bio-phenomenology, as described by Jakob von Uexküll [24]. This was a view held by Husserl, Scheler and Merleau-Ponty, and recognises that in spite of degenerating cognitive abilities the person with dementia continues to experience the world and create meaning, often in affective responses to stimuli e.g. laughing, crying, expressing frustration [7]. Indeed, it has been shown that in people with severe amnesia that an affective response may continue even after the memory of what instigated it has gone [25]. This is supported by the symbolic interactionist view that the self is concealed rather than lost as the dementia progresses [13]. This viewpoint allows carers of people with dementia to provide care beyond the basic physical needs of a person, bestowing meaning to their lives and enriching their daily experience [13].

Ashworth and Ashworth [26] further this notion by stating that we should consider the person as a whole i.e. the person with dementia AND the illness itself in equal measures. They suggest that the person with dementia continues to have a changing inner-life, and that their bodies continue to carry out actions without conscious control (i.e., pre-reflexively) even in the later stages of the illness. This implies that we need to acknowledge the disease process, and that this changes people (therefore not assuming that people stay the same) and how they interact with the world, while at the same time appreciating that the person with dementia is an embodied being who can interact on varying levels with their world even if this is on a pre-reflexive level.

Cohen-Mansfield et al. [27] stated that the sense of *personal self* continues throughout the process of cognitive loss in dementia, even though many other personal and social identities diminish. This is echoed by more recent research into identity of the person with dementia. Caddell and Clare [28] found that the self was dichotomous: some elements of self remained as dementia progressed while other elements changed through the course of the illness, which caused those diagnosed with dementia to feel in a state of flux. This has important connotations for care of people with dementia in that the care giver must be sensitive to the individual’s past and present identity, being mindful of any changes to this so that appropriate support can be offered.

## 4. Personhood

Taking forward this notion of ‘self’, a more contemporary concept of ‘personhood’ has been developed within dementia care. The notion of personhood is not new, and was originally discussed by Rene Descartes (1596–1650) and John Lock (1632–1704). The original perspective was based on the idea that cognition (e.g. the ability to remember past events and actions, self-identity and reason) is essential for personhood to exist [29]. While not a new idea, this altered approach of personhood within dementia care was introduced by Tom Kitwood in the early 1990s as a new way of understanding dementia and providing support in order to maintain personhood [8]. This reconceptualisation of personhood follows a social constructionist approach. That is, personhood is defined as “a standing or status that is bestowed upon one human being by others in the context of particular social relationships and institutional arrangements” [30]. This perspective of personhood promotes a shift from the biomedical model, suggesting that the symptoms (i.e. performance, behaviour) and quality of life of the person with dementia are a result of the social interactions of the person with others rather than a result of the neurological changes [31]. 

Sabat also proposed an alternative view of the self within dementia following the social constructionist view. He proposed that the self has 3 forms: self 1, the singular self; self 2, the physical, mental and emotional characteristics of a person and the beliefs they hold about that; self 3, the publicly presented persona [21,32,33,34]. Sabat stated that the self in all of its forms exist through engagement with others, and that this relies on the cooperation of others within a social context to construct the identity of a person [21]. When a person has dementia, their collaborative attempts to create a particular self with another person may not be successful if the other person refuses to cooperate. This, according to positioning theory, places the person with dementia in a negative position, which can cause misinterpretation and social misunderstandings of their behaviour [21,35]. In terms of dementia care, the social positioning of the person with dementia is important: if negatively positioned, the self of the person is deconstructed to the point of being lost. If the person with dementia is positively positioned and supported, the self is maintained.

Kitwood developed an approach to dementia care which sought to view personhood in social terms, suggesting that the approach of carers was essential to supporting the self of the person with dementia [2,36]. He described the ways in which the actions of caregivers (intentional or unintentional) can have a detrimental effect on the wellbeing of a person with dementia [3]. Kitwood called these negative interactions ‘Malignant Social Psychology’ and he went on to describe 17 types of interaction (or ‘Personal Detractions’) which range in severity and cause a reduction in wellbeing in the person with dementia [2,3,37]. Indeed, Tom Kitwood went on to describe the process of ‘rementia’ which can happen when the personhood of the person with dementia is supported [38]. This approach to the person with dementia can create the phenomenon of temporary improvement or stabilisation in the symptoms of dementia [38]. It has been argued that rementia occurs when changes are made to the personal, social and neurological influences on a person with dementia, and that supporting a person in this way can lead to them regaining some of their lost abilities [20,37,38,39,40,41].

While Kitwood’s approach to dementia care has been widely adopted throughout services, it is not without criticism. Some argue that the approach does not go far enough to encapsulate all of the factors which apply to people with dementia, including citizenship, embodiment, discourse, and spirituality [8,42,43,44]. Additionally, more recent research has suggested that personhood is transient according to the situation of person with dementia, and that people with dementia often strive to retain their sense of personhood in spite of the diagnosis of dementia [45,46]. Indeed, one author went so far as to say they found no evidence of a loss of personhood in people with early stage dementia and supportive caregivers, with little evidence of Kitwood’s ‘malignant social psychology’ and participants who maintained a positive sense of self even after living with dementia for some time [46]. It was acknowledged in this study that those involved had good support networks which potentially preserved the sense of self in the person with dementia. This would imply that Kitwood’s theory of person centred care was correct and that with the correct support a person with dementia can maintain a sense of personhood.

## 5. Embodied Selfhood

Of the criticisms of Kitwood’s notion of personhood, the concept of the embodiment of self is particularly pertinent to the care of a person with dementia. This was proposed by Pia Kontos following a period of research in a specialist Alzheimer’s unit within a Canadian long-term care facility. Kontos proposed that selfhood is not only the consequence of social interactions with others, but it is also the unique way in which our bodies reflexively behave to express our individuality [42]. This was derived from the works of Merleau-Ponty [47] and Bourdieu [48], who argue that our bodies act habitually and pre-reflexively (i.e. without cognitive awareness) to interact with the world. Kontos argued therefore, that people with dementia act in a pre-reflexive manner to express their selves and interact with others, and that this is manifested in the way their body moves [42]. It is argued that embodied selfhood should be made central to the care of a person with dementia, and the intricacies and subtleties of behaviour and action embraced [42]. In doing so, a greater understanding of the ways in which people with dementia remain connected with others through the projection of their selves through non-verbal means would be developed [42]. 

## 6. Conclusions

While the approaches to the self in dementia discussed here are not exhaustive, they represent the current mainstream opinions within dementia care settings within the UK. Historically, the view that the self erodes to nothing or a non-person was the predominant approach, directing care which was not person centred. However, since the advent of the approach proposed by Tom Kitwood, the view has changed to one which recognises that the self and personhood of a person remains and must be supported in order to increase the wellbeing of a person with dementia. Kitwood’s Person-Centred Model of Dementia care has proved to be excellent in advancing the care of people with dementia, however, this is not without flaws, and other approaches should be considered which progress this approach to create supportive, inclusive and understanding care for the person with dementia.
